# Exploration of the Teaching of Mental Health Education Courses in Fine Art Colleges and Universities From the Perspective of Positive Psychology

**DOI:** 10.3389/fpsyg.2022.904731

**Published:** 2022-07-07

**Authors:** Yi Zhang

**Affiliations:** School of Fine Arts, Zhengzhou University, Zhengzhou, China

**Keywords:** teacher ability, student academic performance, identification, self-valued goals, personal importance, self-regulation skills, positive psychology

## Abstract

The study’s objective is to examine the impact of teacher ability on student academic performance, identification (self-valued goals; personal importance), and self-regulation skills. Additionally, the study examines the mediating effect of identification (self-valued goals; personal importance) and self-regulation skills between teacher ability and student academic performance. The data was collected by the teachers at different colleges and universities in China and 341 samples were used to analyze the data through a convenient sampling technique. Moreover, partial least square structural equation modeling is used in data analysis with Smart PLS software. This research indicates that teacher ability has a positive and significant effect on student academic performance, identification (self-valued goals; personal importance), and self-regulation skills. Further, the study also discovers that identification (self-valued goals; personal importance) and self-regulation skills significantly mediate between teacher ability and student academic performance. The study is helpful for the teachers to adopt the better strategies and abilities in themselves when they are engaged with the student in teaching activities.

## Introduction

Internationally, education has become an imperative tool in fostering the growth of individuals, communities, and nations. However, in the 21st century, the high emphasis on technological advancement has made the worldwide educational sector experience massive transformations. The global technological changes have highlighted the need to establish a knowledge-based economy, potentially fostering the nation’s growth (Bal-Taştan et al., 2018). Today, education, regarded as the most profound development, demands the knowledge members (e.g., instructors, learners) to exhibit essential skills and abilities, fundamentally contributing to the country’s economic foundation.

In recent years, education, the product of various resources, has led educational members to play a significant role in driving the knowledge industry. Concerning this, teachers are a vital resource contributing to today’s economy. They hold an authority that encourages them to lead schooling activities with professional abilities. Given the explanation, the research shows that teachers manifesting the leading skills, attributes, and knowledge enables the students to enjoy favorable learning outcomes (Rodriguez and Abocejo, 2018). Teachers’ capabilities improve the students’ learning performance (Arnaiz-Sánchez et al., 2020). However, in contrast, the study shows that previous research has separately focused on teachers’ capabilities and students’ performance. Still, the combined impact of teaching abilities on student performance is yet to be explored (Khan and Ghosh, 2018).

Consistently, the call for effective teaching to favorable student learning outcomes has made teachers’ ability essential for fostering the individual’s performance. Surprisingly, in the context of China, the research shows that approximately 16 million teachers had demand educational training sessions (MOE, 2018). Indeed, the study shows that China’s traditional schooling system has profoundly matured, where the teachers’ functional role in enhancing the educational structure has not significantly altered (Lo and Ye, 2017). Fundamentally, this statement elevates the need for transforming China’s schooling system, which is more conducive to improving student academic performance. Therefore, the People’s Republic of China adopting a modern schooling system requires teachers to exhibit superior learning abilities. In this process, this study calls for the role of teachers’ skills in creating a knowledge economy, thus providing critical guidance for improving students’ performance.

In particular, an improved academic performance demands focusing on purpose identification (i.e., self-valued goals, personal importance), which alludes to individuals’ self-belief in personal goals (Mieghem et al., 2020). In particular, identification refers to accepting the influence, thus creating a valuable relationship (Kelman, 1958). Identification involves valuing the reason behind the action. Identification provides clarity to students, thus helping them pursue the predefined goal. Given the illustration, the study shows that goal identification guides the students to realize the reason behind the goal, thereby improving their learning performance (e.g., academic performance) (Alhadabi and Karpinski, 2020).

Furthermore, self-regulatory skills contribute to achieving the desired goal. Professional learners open to difficulties willingly accept the challenges based on their learned skills. Self-regulatory skills refer to an individual’s belief in obtaining the set goal. It makes individuals regulate their learning activities from a multi-level perspective (e.g., cognition, psychological, behavioral) (Zimmerman and Schunk, 2001). In explaining this notion, the study states that high self-regulatory skills influence the students learning, thus making them perform better than the other students (Lin and Lai, 2019). Indeed, this profound learning competency empowers students to exhibit significant educational traits, potentially enhancing their academic performance.

The Chinese education system demands educationists to adopt novel abilities and skills, thus fostering the student’s performance. However, the teacher-centered approach concerning students’ performance has faced criticism in the prior literature. China is the most goal-oriented country that strongly focuses on improving its educational system. Consistently, the literature shows that various researchers have found difficulty relating teaching abilities with student learning (Khan and Ghosh, 2018). However, despite the increasing significance of high-quality teaching, it has become an open challenge for today’s researchers, practitioners, and policymakers to reach a consensus regarding the specific teaching abilities influencing educational outcomes. Hence, this study set forth the need for rich academic literature explaining the relationship between the teachers’ capabilities and student learning performance. Moreover, the literature showed that the prevailing research gap also calls for a learning process that encourages Chinese students to embrace self–regulation skills. Indeed, this debate paves the way for future studies to adopt alternative approaches (i.e., self-regulation learning), ensuring the continuous development of teachers’ and learners’ capabilities (Nasri et al., 2020).

Significantly, in an attempt to fill the research gap, the study aims to investigate the factors influencing the performance of university students. The study objective is to examine the influence of teaching abilities on student academic performance. Moreover, the study framework determines the impact of self-regulation skills and identification (i.e., self-valued goals, personal importance) on student learning performance. Similarly, the study also highlights the mediating role of identification and self–regulation skills nexus the relationship between teaching abilities and student academic performance.

Profoundly, the paper’s findings potentially contribute to various disciplines. This study is a novel contribution that explores the areas that have remained under-researched. The suggested framework illustrates notable study findings at the high school level, thus providing a refined understanding of the subject. It fundamentally motivates researchers to focus on student performance from the context of teaching capabilities and self-regulation skills. It empowers the students to take forward their learning activities by reflecting on different factors, particularly from the viewpoint of an educationist.

Previously, literature has posed policymakers with a question concerning the impact of teaching capabilities at all school levels. Perhaps, this is a unique study that helps determine the answer to all questions that have previously remained unanswered. Indeed, the answer to these questions provides implications for fostering the efficiency of the teaching process. This study analysis presents a wide range of empirical evidence that helps examine the impact of teachers’ capabilities linked to student performance. The study provides greater clarity on the wisdom of teaching abilities, thus providing implications for future policymakers. Therefore, developing an approach valuing the teachers’ teaching skills based on the evidence may be a promising act for future recommendations and directions.

The study is also beneficial for the policymakers and governments to provide a better facilitation and pleasure environment to the academic sector. This research gives the researchers new insight into developing new literature in this field. Section two explains the study literature review; section three explains the study methodology; section four is about results; section five focuses on the study discussion, while section six explains the study conclusion.

## Literature Review

### Teacher Ability and Student Academic Performance

In the educational sector, the increasing globalization and technological advancement have created a need for superior academic performance. In the 21st century, profound teaching abilities have drastically altered the worldwide educational system, essentially directing the conventional learning methods to competency-based teachings. However, this theoretical foundation of the knowledge economy has extended the student learning perception from traditional coaching to active learning. In today’s digital economy, knowledge transfer from teachers to students has become pivotal for enhancing the individual learning output. Accordingly, the study states that the desire to foster the global education sector encourages teachers to exhibit academic knowledge skills and abilities, thus improving students’ academic performance (Kapur, 2018).

In particular, teaching abilities are an innovative contribution, enhancing the students’ performance. Given the articulation, the research shows that the instructor’s teaching competency is congruent with individuals’ learning outcomes (Bal-Taştan et al., 2018). Significantly, instructors play a vital role in directing learning activities, thus accelerating students’ performance (Bafadal et al., 2018). Undoubtedly, students alone cannot improve their performance. Therefore, the literature requires the teaching profession to facilitate, develop, and maintain academic goals, influencing student learning performance.

Teachers are the fundamental agents promoting the students learning process. Teachers’ abilities (i.e., knowledge, skills, motivation) ensure the implementation of an effective teaching method. It serves as the motivational factor for low-grade achievers, thus boosting their grade performance. Perhaps, the discussion on the effect of teachers’ ability on student performance demands the professionals to critically rely on the instructors’ teaching skills, thereby improving the learning activity (Bakar, 2018). Instructors’ teaching ability influences the students’ educational outcomes. Based on this statement, the study suggests that adopting advanced teaching skills improves students learning performance (Karrera et al., 2019). Therefore, it is vital to polish the teachers’ attributes, thus increasing the effectiveness of professional learning. Hence, the literature concludes that it has become imperative for knowledge members (e.g., teachers, students) to understand the significance of teaching attributes in influencing one’s performance. Therefore, based on the prior research, we have developed the following hypothesis:

H1: *Teacher ability has a positive and significant impact on student academic performance.*

### Teacher Ability and Identification

Teachers significantly contribute to the learning process. They play an instrumental role in improving the effectiveness of the learning system. They work as a facilitator guiding the students’ self-perception regarding goal achievement. Identification is a multidimensional construct that influences students’ choices and actions concerning teachers’ abilities. Given the illustration, the study shows that teachers’ high competency (e.g., belief) strongly relates to student motivation and goal achievement (Lazarides et al., 2018). Teachers ensure developing professionalism that enacts individuals to stay connected to their goals. The goal identification encourages students to realize the reason behind the influence. Accordingly, the study proposes that the diverse teachers’ skills shape the individual’s choices, motivation, and goals, thus recording an increase in their responsiveness (Richardson and Watt, 2018). Indeed, teacher professional competencies are the most crucial solution for realization. In particular, from the teachers’ perspective, the most critical competence is boosting individuals’ self-esteem and belief, substantially guiding students in determining their career goals (Ghorbani et al., 2018; Sarfraz et al., 2022b).

Teachers only not help improve skills but also encourage logical reasoning. The goal identification demands teachers to motivate students, thus boosting their confidence in their actions. In particular, teaching competencies makes the students look at education from a broad perspective. Given the explanation, the research indicates that the teachers’ efficacy, attributes, skills, and competencies stimulate higher students’ commitment, thus enhancing goal value (Donohoo, 2018; Sarfraz et al., 2022c). Indeed, teachers’ beliefs, practices, and attitudes boost the learners’ motivation and achievement. Based on this statement, the study states that teachers’ abilities guide the individual’s sense of efficacy, making them effectively handle the professional activity (Barni et al., 2019). Altogether, based on the current research evidence, the hypothesis states,

H2: *Teacher ability has a positive and significant impact on identification (Self valued goals; personal importance).*

### Teacher Ability and Self-Regulation Skills

Over the past decades, the teaching theories have massively realized the development of the teachers’ abilities, thus supporting students’ academic learning. High-quality teaching capabilities foster the students’ self-learning process. However, today, online education has fundamentally gained teachers’ attention, substantially promoting students’ self-learning. This recently introduced competency has advocated the demand for remote education, significantly shifting traditional learning to self-regulating learning. In particular, coping with such a situation has made the knowledge members (i.e., instructors) face difficulty providing directions. Perhaps, to deal with these emerging challenges, self-regulation skills have been widely embraced as a tool for fostering today’s learning activities. However, concerning an online setting, the study shows that teachers’ facilitation enhances students’ understanding and engagement (Xu et al., 2020), thus promoting the self-learning process.

In a remote setting, self-regulation skills refer to monitoring one’s performance (Li et al., 2018; Sarfraz et al., 2022a). The self-regulation skills drastically influence the teacher’s behavior and students’ motivation. Therefore, to increase academic performance, students need to develop these skills. Self-directed learning makes students develop a positive attitude, thus professionally responding to the profound teaching abilities. Given this, the study shows that in recent years, these competencies have made students actively participate in the learning activity with the support of productive teaching abilities, which is a continuous way of mentoring student learning (Saiyad et al., 2020).

Undoubtedly, gaining this skill may be difficult for the students. Consequently, to produce desired learning outcomes, teachers’ abilities (i.e., motivation, skills, beliefs) help students memorize the learning material by concentrating on their studies (Kapur, 2018). Hence, a teacher holding accountability for improving the students learning outcomes seems simple, but in reality, it is not an easy endeavor. The teaching abilities require the professional educationists to fulfill their obligations, thus planning profound self-learning. However, the enhanced accountability makes the student identify, evaluate, and assess learning goals which may be a challenge without the instructor’s assistance (Kusumaningrum et al., 2019). In conclusion, the hypothesis suggests,

H3: *Teacher Ability has a positive and significant impact on self-regulation skills.*

### Identification and Student Academic Performance

Fundamentally, students’ approach to learning determines their attitude and behavior toward achieving the learning outcome. The prime construct explaining this notion is an individual approach to enhancing the goal orientation. Identification helps individuals understand the reason behind the goal. In support, the work on the development of goal identification states that self-valued goals motivate individuals to exhibit superior competency, thus improving their academic performance (Ngwira et al., 2017). Obtaining good grades with positive learning outcomes encourages self-motivated performance. In particular, the self-perceived emotions of achieving the goal influence the students learning. Given the illustration, the research shows that student efficacy enhances the learning process, leading to improved learning performance (Hayat et al., 2020).

Significantly, students are judged based on their annual performance. Literature shows that several components influence the students’ outcomes. Out of which, personal values and self-determination lead the student to experience potential learning. However, mastery over the goals demands students to develop performance goals for accelerating their academic output. Hence, to gain favorable learning outcomes, a performance approach orientation (i.e., self-perceived goal) ensures an improvement in student performance (Scherrer et al., 2020).

Additionally, student academic self-belief ensures the successful fulfillment of the learning task. This self-belief makes an individual exhibit superior performance, commitment, and endeavor. The increased identification makes the students identify the reason behind the work task, thus minimizing their chances of failure. However, in contrast, students low on identification face difficulty understanding the work task. In explaining this phenomenon, a study performed on 214 university students revealed that students believing in the work task produce a significant educational outcome (Alyami et al., 2017; Sarfraz et al., 2021). Furthermore, in line with the current statement, the study also shows that students’ academic confidence increases their learning, motivation, and educational performance (Doménech-Betoret et al., 2017). Hence, based on the previous findings, the hypothesis proposes,

H4: *Identification (Self valued goals; personal importance) has a positive and significant impact on student academic performance.*

### The Mediating Role of Identification

Significantly, in recent years, global educational changes have transformed the traditional teaching methods into remote learning, thus elevating the need for understanding the concept of identification. Self-valued goals help students interpret and evaluate the reason behind their actions. Perhaps, concerning this, teachers’ skills play a vital role in making the work meaningful for students. In explaining this notion, the study indicates that improved performance requires teachers to expand their professional competencies, substantially helping the students achieve their learning goals (Kalimullina and Trotsenko, 2018).

Altogether, the concept of identification (i.e., personal importance) provides the individual with a meaningful learning experience concerning the learning activities and event. The teachers’ competence elevates the professional status of the work activity, thus making work meaningful for students. Given the explanation, the study states that the manifestation of teachers’ skills and abilities makes the student realize the value of the learning activity (Kostiainen et al., 2018), substantially improving the academic outcome. Perhaps, this research makes understanding the learning process from the teacher-student construct vital for today’s education members.

In particular, the teaching competency supports the students learning process. Accordingly, professional engagement and self-management are the significant dimensions of teachers’ capabilities. Each of these competencies allows the students to find the value of their goal through professional help and assistance. Teachers establish an effective learning process, thus managing the students’ activities. Given the explanation, the study states that teaching abilities positively influence individuals’ knowledge by significantly directing them to achieve favorable learning outcomes (Manasia et al., 2019).

Undoubtedly, identification is strongly related to teachers’ abilities where developing professionalism is the foremost responsibility of teachers. As a result, many scholars have supported the notion that student learning is highly dependent on teaching abilities. In supporting this statement, the research indicates that teachers’ professionalism works as a strategic driver that enhances the individual self-belief, work status, and sustainable education performance (Wolff et al., 2017). Indeed, the profound concept of learning (i.e., identification) makes teachers a significant player in improving student learning results. Consequently, based on the previous literature findings, we have proposed the following hypothesis:

H4(a): *Identification (Self valued goals; personal importance) mediates the relationship between teacher ability and student academic performance.*

### Self-Regulation Skills and Student Academic Performance

Significantly, education is a strategic tool ensuring the growth of individuals, communities, society, and nations. Therefore, to fully benefit from this, innovative educational methods need to be highly embraced to ensure individual progress. Learning skills play an imperative role in improving students’ academic performance. In particular, self-regulation skills are an integrated concept for promoting academic performance. It helps students gain mastery over their learning activities, thus achieving their career goals (Askar, 2018). Indeed, it is a profound factor determining the students’ performance.

The self-regulation skills work as a tool, enhancing students’ confidence, thus supporting superior academic results. In the illustration, the study shows that the self-regulation construct strengthens students’ performance and goals (Duckworth et al., 2019). Self-regulation skills enable individuals to manage their emotions and behavior, thus ensuring positive academic performance. In a learning environment, student self-regulation skills enhance their ability to improve academic results. It inspires students to focus on studying with complete mindfulness. Perhaps, it empowers individuals to create and enjoy a sustainable academic performance by massively adopting novel learning methods. In explaining this, research shows that students’ self-regulatory skills are vital for achieving academic success (Rooij et al., 2018). In particular, the literature demands educational members to adopt transformative skills for coping with the increasing academic demand (Ghorbani et al., 2018). According, student needs to develop competencies that support their learning concept. Indeed, the literature concludes that attention to the students’ self-directed competency is vital for improving one’s performance. Therefore, in response to the previous literature, our hypothesis concludes:

H5: *Self-Regulation Skills have a significant and positive impact on Student Academic Performance.*

### The Mediating Role of Self-Regulation Skills

Educational policymakers and researchers are increasingly interested in studying the role of teachers’ abilities in influencing the student’s learning process. In particular, instructors’ competencies provide learners with valuable feedback that helps them analyze the value of critical information, enhancing their learning outcomes. Instructors’ feedback makes students identify their learning goals, thus providing a clear understanding of their work task. In particular, teachers’ feedback plays a strategic role in boosting individuals’ self-management qualities. The result shows that teachers’ collaboration directs the students’ actions, and learning activities, thus influencing their academic performance (Muenks et al., 2018).

In addition to providing feedback, teachers also guide the learners in achieving goals based on their capabilities. Teachers play a vital role in directing self-regulation skills. The teacher increases confidence in individuals, thereby encouraging obtaining improved academic results. Teachers’ professional knowledge encourages students to embrace novel tools for achieving favorable learning outcomes (Manasia et al., 2019). Undoubtedly, superior teaching abilities work as the catalyst, enhancing the students’ performance. It motivates the students to polish their skill set, eventually gaining positive learning results. Given the explanation, the study shows that positive teaching encourages students to adapt to self-directed learning, thus facilitating better academic grades (Khan and Ghosh, 2018).

Indeed, self-learning is an excellent phenomenon that makes student improve their performance. Self-based learning has proven to be an effective learning process boosting student confidence in learning outcomes. This self-direct learning enhances the students’ academic skills, motivation, and efficacy at different levels, grades, and age groups. Given the articulation, the research shows that project-based learning has made teachers increase student confidence, thus making them outperform in their academic courses (Shin, 2018). Based on the empirical evidence, we have developed the hypothesis as follows,

H5(a): *Self-Regulation Skills mediate the relationship between teacher ability and student academic performance.*

**FIGURE 1:**
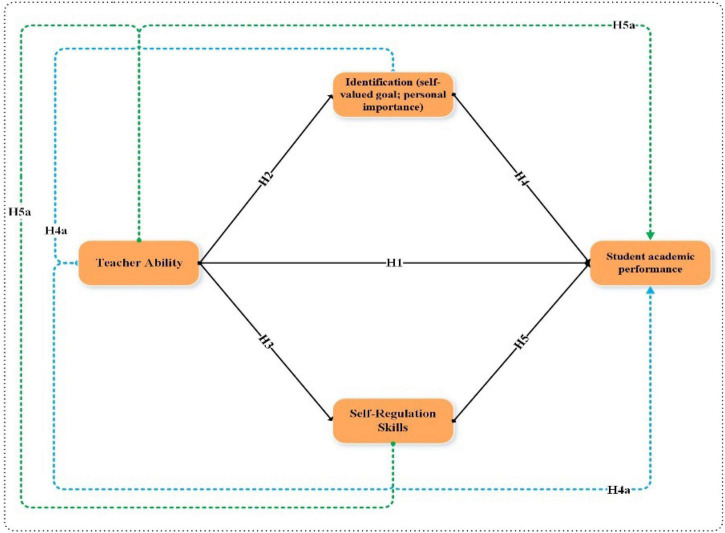
Study conceptual framework.

Figure 1 shows the study hypothesis relationship between independent, dependent, and mediating variables.

## Methodology

The study used the quantitative research to examine the impact of teacher ability on student academic performance, identification (self-valued goals; personal importance), and self-regulation skills. Additionally, the study examines the mediating effect of identification (self-valued goals; personal importance) and self-regulation skills between teacher ability and student academic performance. The study utilized the positivism philosophy, which means data gained by scientific observations and measurements. The deductive reasoning approach is applied in this study. The data was collected from the teachers working in the Colleges and Universities of China. The study adopted the convenient sampling technique for the data collection process. The adopted structured questionnaires were used in data collection.

Moreover, the five Likert scales were used to measure the survey, ensuring the reliability and validity of items. However, before sending the questionnaires to respondents, a cover letter is attached to ensure the confidentiality of respondents for research objectives. The sample of 450 was distributed to the teachers at different colleges and universities in China, and data was collected electronically. After that, 410 questionnaires were received, and final 341 questionnaires were chosen to analyze the data with an 83% response rate. The study used the smart PLS for the Partial least square’s structural equation model. The measure assessment model was used to measure the mediation relationship between constructs. The partial least square and structural equation model can easily test the complex relationship between construct with the use of regression analysis and it is not restricted to the large sample size or data normality (Hair Sarstedt et al., 2014). Teacher ability three items scale was adopted from the study of Alexander et al. (2020). Identification five items scale was a adopted from the study of Ryan and Connell (1989). Self-regulation skills four items scale was a adopted from the study of Lai et al. (2022) while Student Academic Performance 11 items scale was measure from the study of DuPaul et al. (1991).

## Results

Table 1 provides the detail of the demographic characteristics of respondents. In this study, 172 male and 169 female respondents participated. Respondent’s age between 18 and 20 are 13.8%, age between 21 and 23 are 35%, age between 24 and 26 are 36%, age between 24 and 26 are 36%, respondents have age between 27 and 30 are 14%. In education section, 27 have intermediate education 7.9%, 142 respondents have bachelor’s degree 41%, 140 respondents have master’s degrees 41%, moreover 32 respondents are MPhil/others with 9.4%.

**TABLE 1:** Demographic characteristics.

Items	Frequency (*N* = 341)	(%)
*Gender*		
Male	172	50.4
Female	169	49.6
*Age*		
18–20	47	13.8
21–23	122	35.8
24–26	124	36.4
27–30	48	14.1
*Education*		
Intermediate	27	7.9
Bachelor	142	41.6
Master	140	41.1
MPhil/Others	32	9.4

### Common Method Bias

This research also applied the common method bias using Harman’s single-factor approach. The variance extracted by one single factor is 10.089% which is less than 50%, indicating that there is no common method bias in this study (Podsakoff et al., 2003).

### Assessment of Measurement Model

Table 2 shows the reliability and validity analysis. Reliability can be measured through the values of Cronbach alpha and composite reliability. Internal consistency of data is measured by Cronbach alpha, and the predictor of internal consistency is measured by the composite reliability (Hair Sarstedt et al., 2014). According to the criteria, the Cronbach alpha and composite reliability values should be higher than 0.70. All values show the desire for internal consistency and predictivity in this study. AVE is the average variance extracted; those values should be higher than 0.5; compared to the threshold, all values are greater than 0.50.

**TABLE 2:** Reliability and validity analysis.

Construct	Items	Loading	α	CR	AVE
Teacher ability	TA_1	0.748	0.783	0.783	0.547
	TA_2	0.767			
	TA_3	0.702			
Identification	ID_1	0.680	0.846	0.846	0.524
	ID_2	0.704			
	ID_3	0.722			
	ID_4	0.714			
	ID_5	0.793			
Self-regulation skills	SRS_1	0.693	0.822	0.823	0.538
	SRS_2	0.713			
	SRS_3	0.743			
	SRS_4	0.782			
Student academic performance	SAP_1	0.730	0.867	0.866	0.566
	SAP_10	0.844			
	SAP_11	0.746			
	SAP_2	0.811			
	SAP_3	0.717			
	SAP_4	0.740			
	SAP_5	0.766			
	SAP_6	0.706			
	SAP_7	0.643			
	SAP_8	0.775			
	SAP_9	0.723			

**FIGURE 2:**
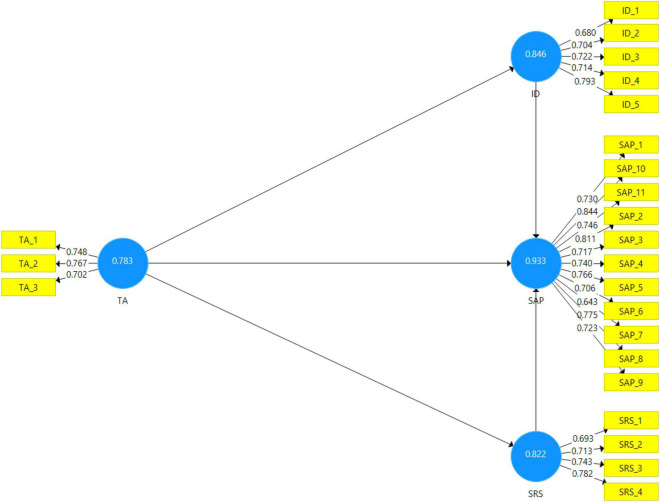
Graphical representation of assessment of measurement model.

Figure 2 displays the graphic illustration of the assessment of the measurement model.

Table 3 shows discriminant validity analysis, which was measured by two methods. One is Fornell–Larcker, and the second is Heterotrait–Monotrait (HTMT). Fornell–Larcker demonstrates the square root value of average variance extracted that should be higher than off-diagonal values, showing the discriminant validity (Fornell and Larcker, 1981). Heterotrait–Monotrait (HTMT) shows the discriminant validity between constructs that values should be less than 0.85 (Henseler et al., 2015). The study indicates that all values are less than 0.85 and established the discriminant validity as per the threshold criteria.

**TABLE 3:** Discriminant validity analysis (Fornell-Larcker and HTMT).

Constructs	1	2	3	4
1. Identification	0.724	0.661	0.648	0.627
2. Student academic performance	0.663	0.747	0.626	0.636
3. Self-regulation skills	0.647	0.626	0.734	0.686
4. Teacher ability	0.629	0.638	0.687	0.740
				

Table 4 indicates (VIF) variance influence factor that demonstrates the multicollinearity between variables and confirms how many constructs influenced the dependent variable. The study results confirmed that all variables have multicollinearity between each other.

**TABLE 4:** Variance influence factor.

Constructs	1	2	3	4
1. Identification		1.936		
2. Student academic performance				
3. Self-regulation skills		2.216		
4. Teacher ability	1	2.132	1	

### Structural Model

Table 5 indicates the hypotheses testing direct effects between variables. The value of beta coefficients, *t*-value, and *p*-value was applied to confirm the acceptance and rejection of hypotheses. The beta values indicate the strength of variables that should be close to the +1 for strong relation (Hair Sarstedt et al., 2014; Hair Hult et al., 2016). Furthermore, the *t*-value and *p*-values show the supported and non-supported hypotheses, the *t*-value should be greater than 1.96, and the *p*-value should be less than 0.05. The study proposed seven hypotheses. H1 is accepted as a teaching ability that has a positive and significant impact on student academic performance. The beta value is 0.268, and the *t*-value is 2.96. H2 is accepted as a teacher’s ability to have a positive and significant impact on identification (Self valued goals; personal importance) with a beta value of 0.629 and a *t*-value of 9.953. H3 is accepted as a teacher’s ability to positively and significantly impact self-Regulation skills, with a beta value of 0.687 and *t*-value of 11.594. H4 proposed that Identification (Self valued goals; personal importance) has a positive and significant impact on student academic performance that is accepted with a beta value of 0.358 and an at-value of 4.633. Moreover, H5 proposed that self-regulation skills have a significant and positive impact on student academic performance is accepted with a beta value of 0.21 and *t*-value of 2.383.

**TABLE 5:** Hypotheses testing direct effect.

Hypothesis	Direct relationships	Std. *Beta*	Std. error	*T* values	*P*-values
H1	TA → SAP	0.268	0.091	2.96	**
H2	TA → ID	0.629	0.063	9.953	***
H3	TA → SRS	0.687	0.059	11.594	***
H4	ID → SAP	0.358	0.077	4.633	***
H5	SRS → SAP	0.21	0.088	2.383	*

*Indicates significant paths: *p < 0.05, **p < 0.01, ***p < 0.001.

**FIGURE 3:**
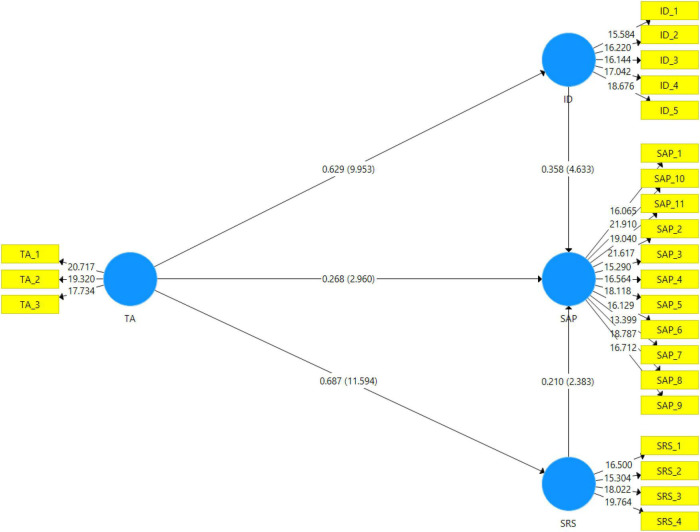
Graphical representation of the structural model.

Table 6 indicates the mediating relationship between study variables. The study proposed that H4a identification (Self valued goals; personal importance) mediates the relationship between teacher ability and student academic performance, accepted with a beta value of 0.144 and 2.246. Further, H5a proposed that self-regulation skills mediate the relationship between teacher ability and student academic performance accepted with a beta value of 0.225 and a *t*-value of 3.986. Figure 3 shows the pictorial representation of the structural equation model.

**TABLE 6:** Hypotheses testing mediation effect.

Hypothesis	Indirect relationships	Std. *Beta*	Std. error	*T values*	*P*-values
H4a	TA → SRS → SAP	0.144	0.064	2.246	*
H5a	TA → ID → SAP	0.225	0.057	3.986	***

*Indicates significant paths: *p < 0.05, ***p < 0.001.

The following Table 7 indicates the quality of the model. *R*^2^ and adjusted *R*^2^ show a strong association between variables. In this study, *R*^2^ values of ID, SAP, and SRS are 0.396, 0.54, 0.472, respectively, and adjusted *R*^2^ values of ID, SAP, and SRS are 0.394, 0.536, and 0.471, respectively. Further, another criterion for the quality of the model is Q^2^ which indicates the predictive relevance of the model. It should be higher than 0 (Hair Sarstedt et al., 2014). Q^2^ values of ID, SAP, and SRS are 0.16, 0.272, and 0.196, respectively.

**TABLE 7:** Quality criteria.

Latent variables	*R* ^2^	*R* ^2Adj^	Q^2^	*F* ^2^
ID	0.396	0.394	0.16	
SAP	0.54	0.536	0.272	
SRS	0.472	0.471	0.196	
ID → SAP				0.144
SRS → SAP				0.043
TA → ID				0.656
TA → SAP				0.073
TA → SRS				0.895

Moreover, the value of *F*^2^ shows the effect size between variables, which means higher *F*^2^ values indicate a larger effect size. In this study, the values of *F*^2^ are 0.144, 0.043, 0.656, 0.073, and 0.895, respectively.

## Discussion

Research indicates that teacher ability has a positive effect on student academic performance. Teachers must believe in their abilities to portray themselves as role models that positively affect the student’s perception and performance. The study supported hypothesis H1, which states that teacher ability has a positive and significant impact on student academic performance. The study supported by teachers is the fundamental agent promoting the students learning process. Teachers’ abilities (i.e., knowledge, skills, motivation) ensure the implementation of an effective teaching method. Teaching abilities are an innovative contribution, enhancing the students’ performance. Teaching competency is congruent with individuals’ learning outcomes (Bal-Taştan et al., 2018). The H2 is supported that teacher ability has a positive and significant impact on identification (Self valued goals; personal importance). The study aligned with the teachers to develop professionalism that enables individuals to stay connected to their goals. The goal identification encourages students to realize the reason behind the influence. Accordingly, the study proposes that the diverse teachers’ skills shape the individual’s choices, motivation, and goals, thus recording an increase in their responsiveness (Richardson and Watt, 2018). Goal identification demands teachers to motivate students, thus boosting their confidence in their actions. The research indicates that the teachers’ efficacy, attributes, skills, and competencies stimulate higher students’ commitment, thus enhancing goal value (Donohoo, 2018). The H3 is supported by teacher ability has a positive and significant impact on self-regulation skills. The findings of this study are supported by literature that self-regulation skills have been widely embraced as a tool for fostering today’s learning activities. However, concerning an online setting, the study shows that teachers’ facilitation enhances students’ understanding and engagement, thus promoting the self-learning process (Xu et al., 2020). Self-regulation skills drastically influence the teacher’s behavior and students’ motivation. Therefore, to increase academic performance, students need to develop these skills. Self-directed learning makes students develop a positive attitude, thus professionally responding to the profound teaching abilities (Saiyad et al., 2020). The H4 is supported that identification (Self valued goals; personal importance) has a positive and significant impact on student academic performance. The results of this study confirmed by self-perceived emotions of achieving the goal influence the students learning. The research shows that student efficacy enhances the learning process, leading to improved learning performance (Hayat et al., 2020). Personal values and self-determination lead the student to experience potential learning. Therefore, to gain favorable learning outcomes, a performance approach orientation (i.e., self-perceived goal) ensures an improvement in student performance (Scherrer et al., 2020). Further, H4a is supported in this study identification (Self valued goals; personal importance) mediates the relationship between teacher ability and student academic performance. The outcomes of the study aligned with previous literature that identification is strongly related to teachers’ abilities where developing professionalism are the foremost responsibility of teachers. As a result, many scholars have supported the notion that student learning is highly dependent on teaching abilities (Wolff et al., 2017). Professional engagement and self-management are the significant dimensions of teachers’ capabilities. Teachers establish an effective learning process, thus managing the students’ activities. Given the explanation, the study states that teaching abilities positively influence individuals’ knowledge by significantly directing them to achieve favorable learning outcomes (Manasia et al., 2019). The H5 is supported self-regulation skills have a significant and positive impact on student academic performance. This study confirmed that student self-regulation skills enhance their ability to improve academic results. It inspires students to focus on studying with complete mindfulness. Perhaps, it empowers individuals to create and enjoy a sustainable academic performance by massively adopting novel learning methods (Rooij et al., 2018). Moreover, H5a is supported self-regulation skills that mediate the relationship between teacher ability and student academic performance. The findings of this study supported by teachers play a vital role in directing self-regulation skills. The teacher increases confidence in individuals, thereby encouraging obtaining improved academic results (Manasia et al., 2019).

## Conclusion

In recent years, Chinese education has considerably captured the attention of researchers and policymakers. Perhaps, in this context, this paper is a unique contribution to Chinese education. The literature highlights the role of teachers’ abilities (i.e., beliefs, motivation, skills) in guaranteeing superior academic performance. In particular, this work aims to improve the student school performance *via* potential teaching capabilities. Consequently, it is a significant study presenting a framework of different factors (e.g., self-regulation skills and identification) stimulating students’ academic performance.

Indeed, the compelling research findings weight the various studies by incorporating variables that statistically elevate student performance. The positive effect of these variables promotes supportive results. The study findings revealed a positive and significant relationship between teachers’ ability, student academic performance, identification, and self-regulation skills. Furthermore, it also indicated a significant mediating effect of self-regulation skills and identification on student performance. Indeed, the study results have supported the previous empirical assumption, thus encouraging us to accept all the hypotheses. Significantly, in the same vein, the study findings present potential implications for education policymakers, researchers, and knowledge members (i.e., teachers) to embrace superior teaching abilities for directing individuals’ learning process, motivation, and performance. The study suggests adopting teaching capabilities as the most fundamental tool for enhancing the individual learning outcome. The article encourages promoting students’ self-regulatory learning, thus improving students’ academic performance.

## Data Availability Statement

The raw data supporting the conclusions of this article will be made available by the authors, without undue reservation.

## Ethics Statement

Ethical review and approval was not required for the study on human participants in accordance with the local legislation and institutional requirements. The patients/participants provided their written informed consent to participate in this study.

## Author Contributions

The author confirms being the sole contributor of this work and has approved it for publication.

## Conflict of Interest

The author declares that the research was conducted in the absence of any commercial or financial relationships that could be construed as a potential conflict of interest.

## Publisher’s Note

All claims expressed in this article are solely those of the authors and do not necessarily represent those of their affiliated organizations, or those of the publisher, the editors and the reviewers. Any product that may be evaluated in this article, or claim that may be made by its manufacturer, is not guaranteed or endorsed by the publisher.
